# Supplementary material for: Surplus fatty acid synthesis increases oxidative stress in adipocytes and induces lipodystrophy
Source: Nat Commun. 2024 Jan 2;15:133. doi: 10.1038/s41467-023-44393-7 (PMC10761979; doi:10.1038/s41467-023-44393-7)
Supplement: Supplementary file 1 — Supplementary Information [file 41467_2023_44393_MOESM1_ESM.pdf]

Supplementary Figure 1

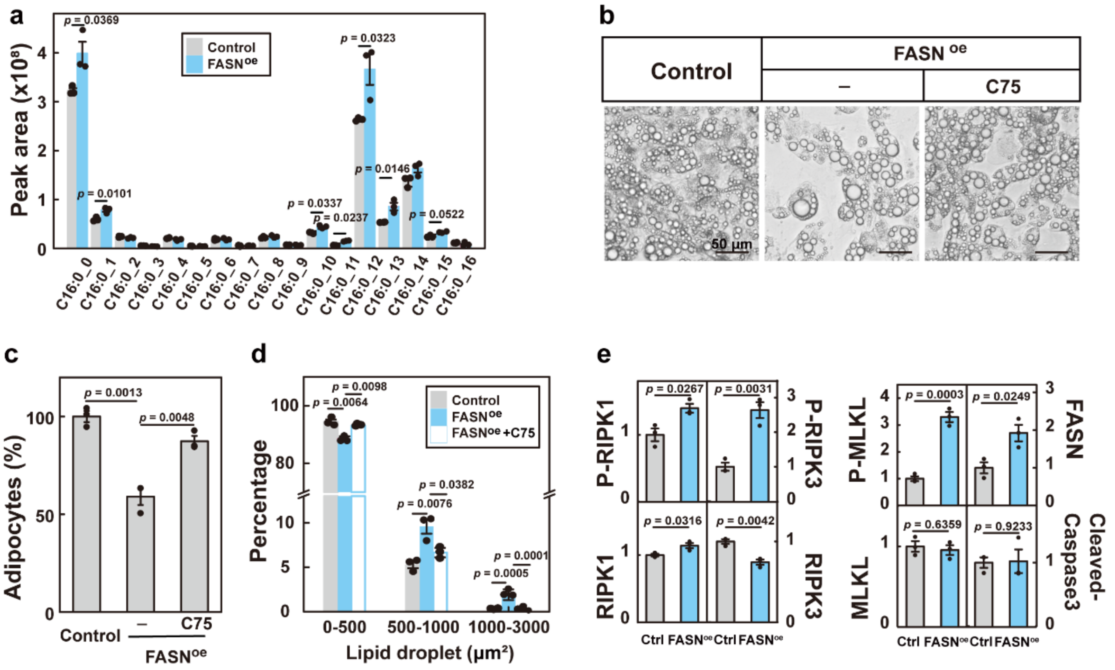

Supplementary Figure 1. Overexpression of FASN leads to necroptosis of the adipocytes. a, On day 10, *de novo* fatty acid synthesis was performed as in Fig. 1e. An illustration of the ^{13}C incorporation into palmitate (C16:0) was shown. **b-d**, Control and FASN^{oe} 3T3-L1 cells were set up and differentiated into adipocytes as in Fig. 1h. Starting from day 9, cells were treated with C75 (50 μM). On day 15, cells were harvested for imaging under bright field (b), quantification of the numbers of adipocytes (c) and lipid droplet size (d). **e**, Quantitative analysis of the protein levels in Fig. 1o. For a, c, d and e, each value represents mean ± s.e.m. a triplicate. Statistical analysis was performed using two-sided unpaired Student's t-tests.

Supplementary Figure 2

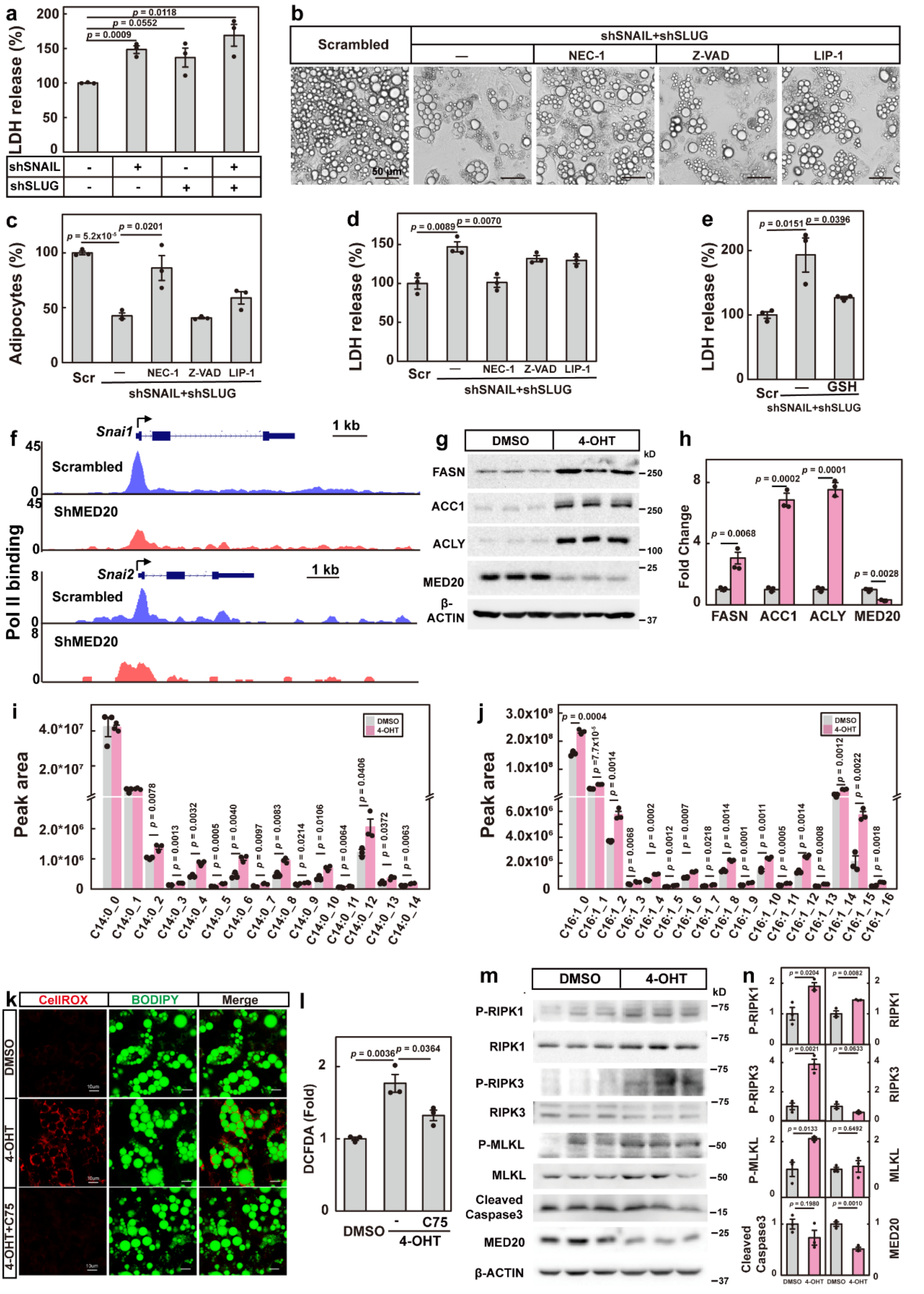

Supplementary Figure 2. MED20 inhibits the transcription of *Fasn* through SNAIL and SLUG. a, The experiment was set up as in Figure 3b. The released LDH were

measured. **b-d**, Starting from day 9 of differentiation, SNAIL and SLUG double knockdown adipocytes were treated with NEC-1 (20 μM), ZVAD (10 μM) or LIP-1 (200 nM). On day 15, cells were harvested and subjected to imaging under bright field (**b**), quantification of cell numbers (**c**) and released LDH (**d**). **e**, The experiment was set up as in Fig. 3d. The released LDH were measured. **f,** ChIP-Seq analysis of the binding of Pol II on the promoters of *Snai1* and *Snai2* in control and MED20 knockdown 3T3-L1 cells. These data were reanalyzed from GEO: GSE163281. **g,h,** Control and MED20-depleted (4-OHT) adipocytes were harvested on day 9 of differentiation. Cells were subjected to western blot using the indicated antibodies (**g**) and quantification of the protein levels (**h**). **I,j**, An illustration of the incorporation of ^{13}C into myristate (C14:0) (h) and palmitoleate (C16:1) (i). The experiment was as performed in Fig. 3i. **k**, The experiment was set up as in Fig 3j. Representative images were shown. **l**, On day 12, cells were treated with DCFDA (25 μM) for 45 min and harvested for quantification of the fluorescent intensity. **m,n,** On day 10 of differentiation, Control and MED20-depleted (4-OHT) adipocytes were harvested and subjected to western blot using indicated antibodies (**m**) and quantification of protein level (**n**). For h and n, β-actin was used as an internal control. For a, c, d, e, h, I, j, l and n, each value represents mean ± s.e.m. a triplicate. Statistical analysis was performed using two-sided unpaired Student's t-tests.

Supplementary Figure 3

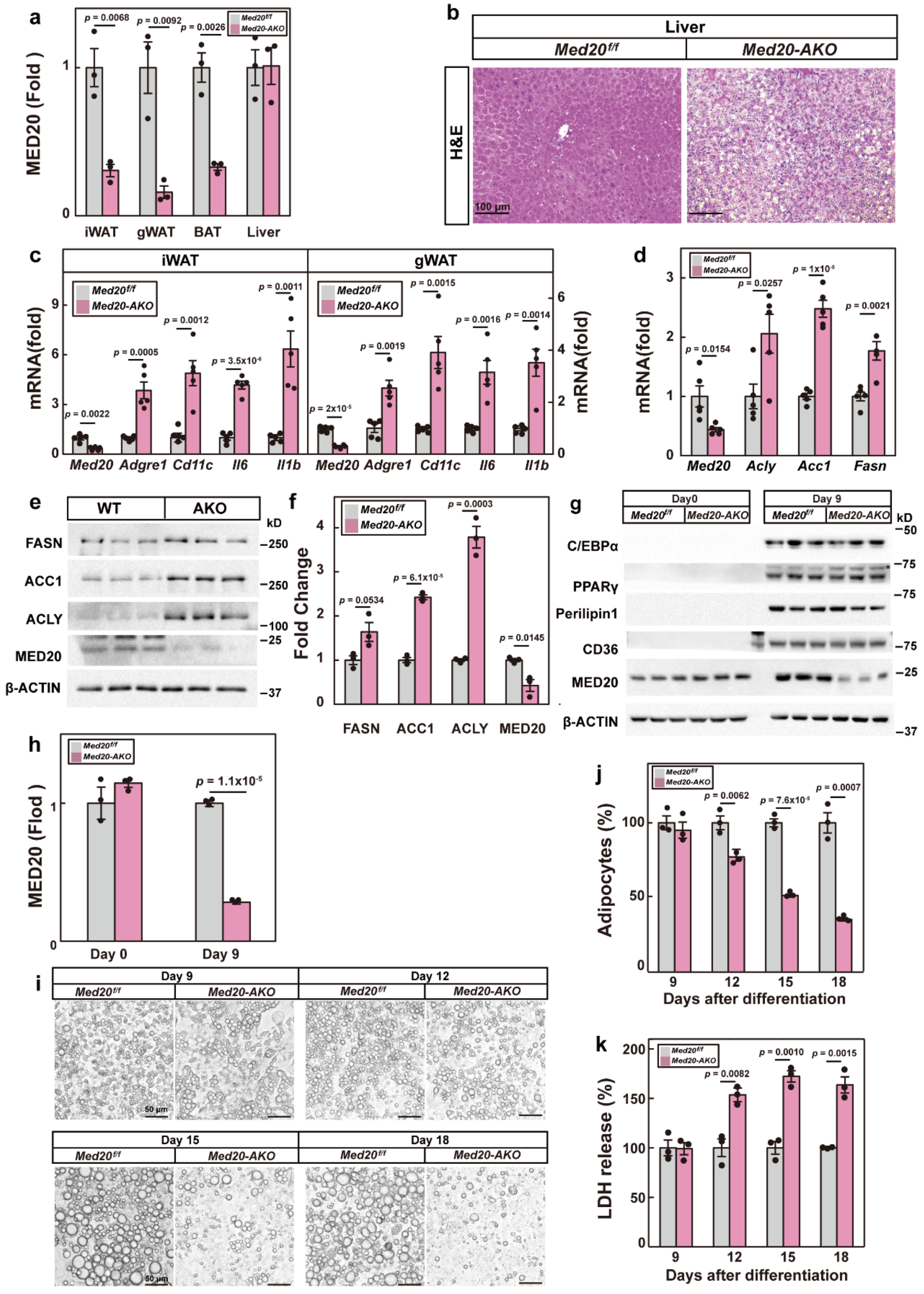

Supplementary Figure 3. Knockout of *Med20* in adipose tissues leads to lipodystrophy. The same mice were from Fig. 4b. **a**, The protein level of Fig 4a was

quantified. **b**, H&E analysis of liver samples. **c**, qRT-PCR analysis of the inflammation-related genes in iWAT and gWAT. 36B4 was used as an internal control. **d-f**, RNA (**d**), protein (**e**) analysis of *de novo* fatty acid synthesis genes in iWAT. The protein level in (e) was quantified and shown in (f). Each value represents mean ± s.e.m. of 3-4 mice. **g-k,** Primary SVFs were isolated from *Med20*$^{f/f}$ or *Med20-AKO* mice and differentiated into mature adipocytes. On the indicated days, cells were harvested for western blot (g,h), imaging under bright field (i), and quantification of cell number (j) and released LDH (k). For a, f and h, β-actin was used as an internal control. For h, j and k, each value represents mean ± s.e.m. a triplicate. Statistical analysis was performed using two-sided unpaired Student's t-tests.

Supplementary Figure 4

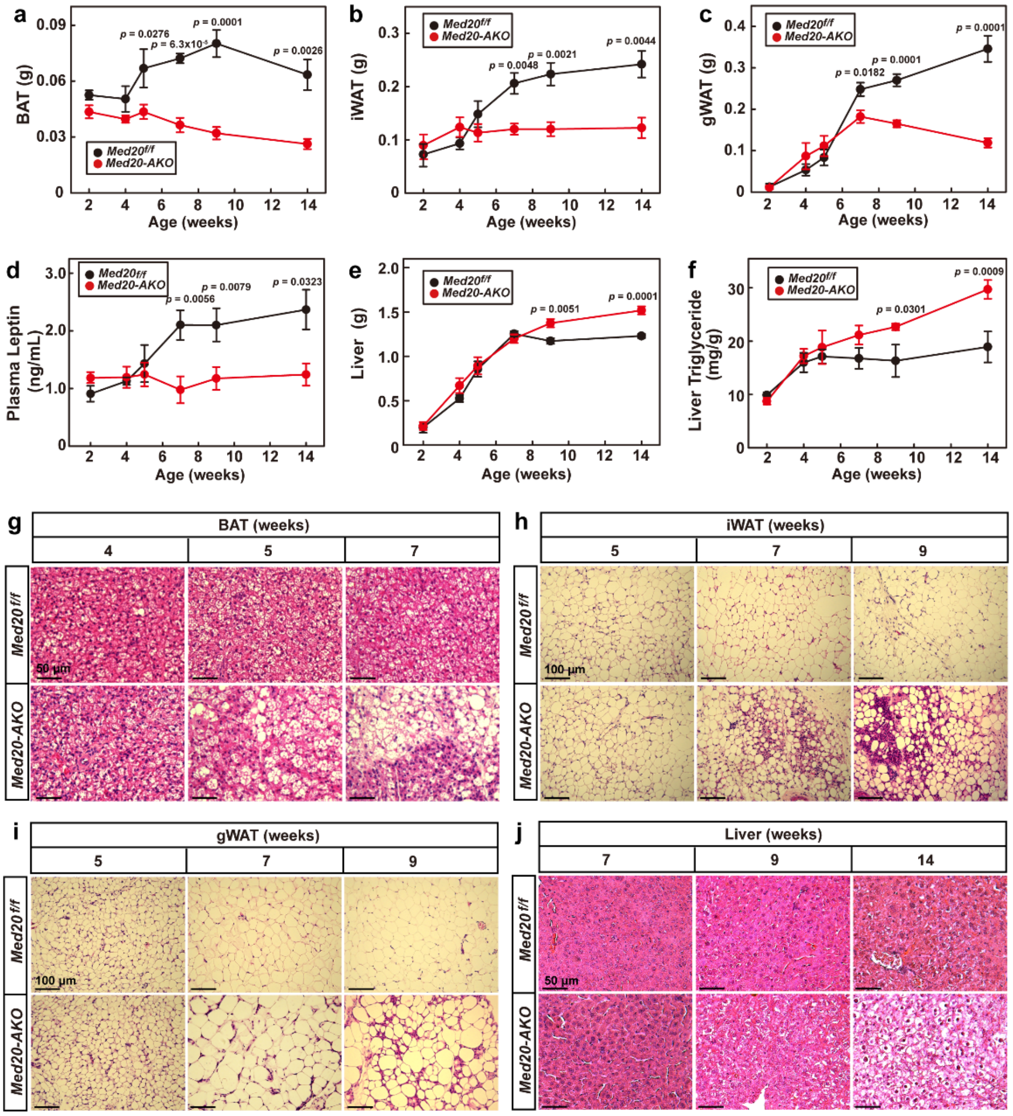

Supplementary Figure 4. *Med20-AKO* mice progressively develop lipodystrophy.

a-j, Chow-fed *Med20*$^{f/f}$ and *Med20-AKO* mice (male, 4-6 per group) were examined at different ages. Weights of BAT (a), iWAT (b), gWAT (c) and liver (e), plasma leptin (d) and liver triglyceride (f) were measured. H&E staining of BAT (g), iWAT (h), gWAT (i) and liver (j) were performed as in Fig. 4j. Scale bars were as indicated. Each value represents mean ± s.e.m. of 4-6 mice. Statistical analysis was performed using two-sided unpaired Student's t-tests.

Supplementary Figure 5

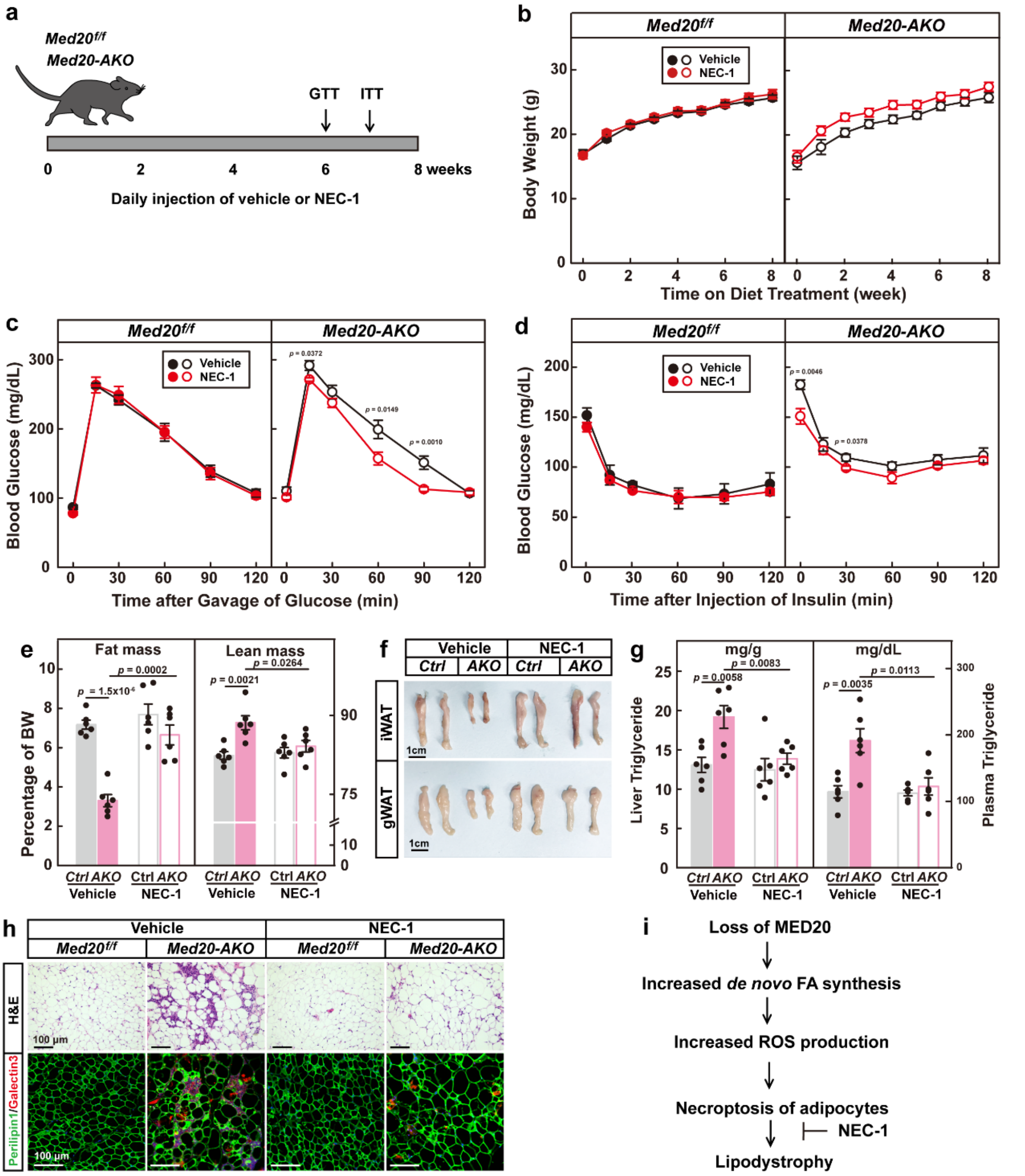

Supplementary Figure 5. Inhibiting necroptosis reverses lipodystrophy in *Med20-AKO* mice. a-h, Chow-fed male *Med20$^{f/f}$* and *Med20-AKO* mice (5-week-old, n=6) were intraperitoneally injected with vehicle (0.2% DMSO in PBS) or NEC-1 (1.65 mg/kg in PBS) every day for 8 weeks. **b**, Body weights were monitored for 8 weeks. **c-d**, Glucose (c) and insulin (d) tolerance tests were performed on week 6 and 7,

respectively. **e**, Body composition was analyzed on week 8. **f-h**, On week 8, mice were euthanized, and tissues were collected. Representative images of iWAT and gWAT were shown (f). Plasma and liver triglyceride levels were measured (g). h, iWAT was subjected to H&E and immunostaining using anti-Perilipin and Galectin3 antibodies. i, A schematic summary of the key findings in this figure. Each value represents mean ± s.e.m. of 6 mice. Statistical analysis was performed using two-sided unpaired Student's t-tests.

Supplementary Figure 6

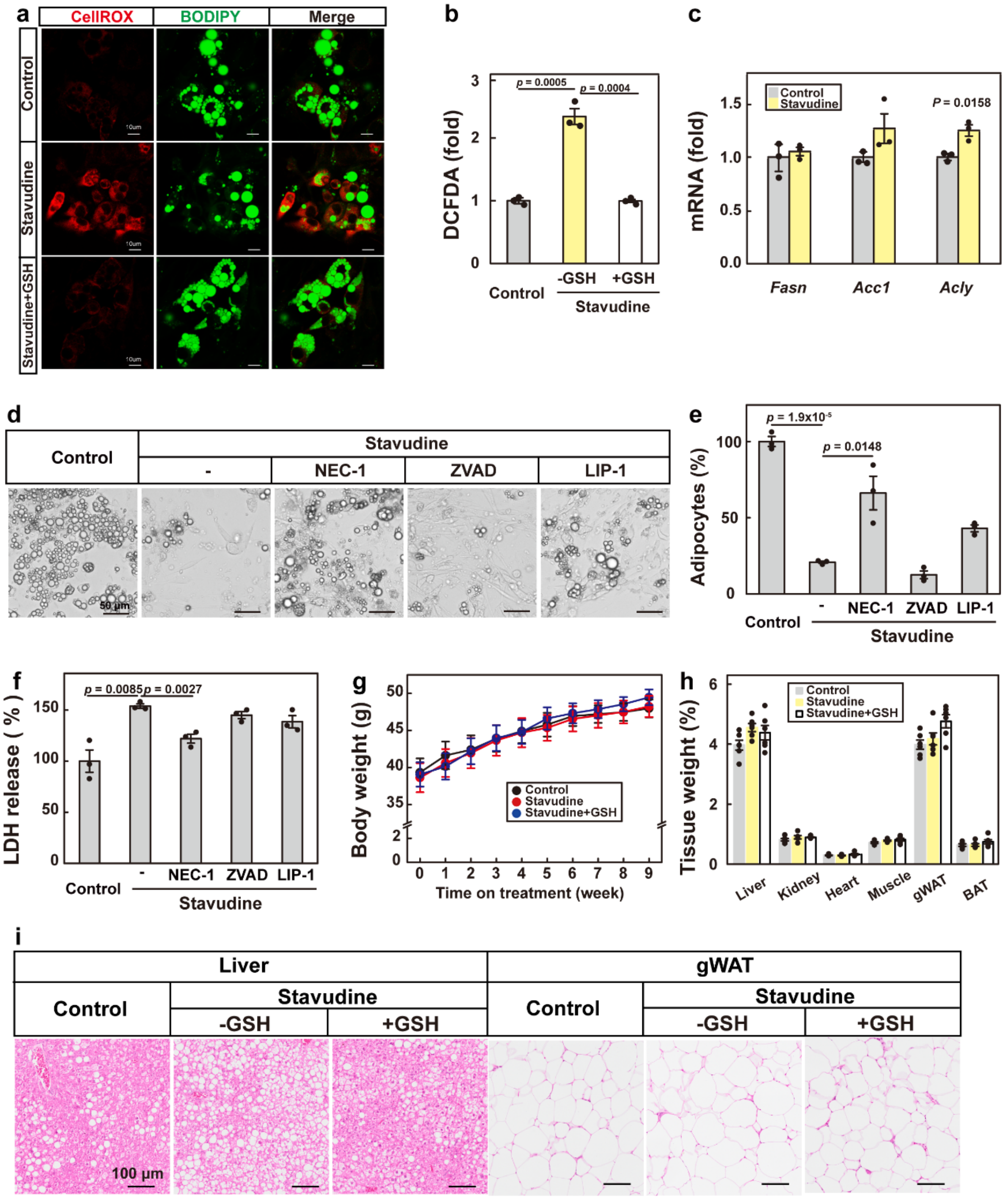

Supplementary Figure 6. Glutathione reverses stavudine-induced partial lipodystrophy in mice. a, Representative images of Fig. 6c were shown. **b**, cells were differentiated into mature adipocytes. On day 0, cells were treated with stavudine (1 mM) with or without GSH (1 mM). On day 4, cells were treated with DCFDA (25 μM) for 45 min and harvested for quantification of the fluorescent intensity. **c**, On day 0,

cells were treated with stavudine (1 mM). On day 4, cells were harvested for qRT-PCR analysis of *de novo* fatty acid synthesis genes. 36B4 was used as an invariant control. **d-f**, 3T3-L1 cells were differentiated into mature adipocytes. On day 0, cells were treated with stavudine (1 mM) in the presence or absence of NEC-1 (20 μM), ZVAD (10 μM) or LIP-1 (200 nM). On day 6, cells were harvested for imaging under bright field (d) and quantification of cell numbers (e) and released LDH (f). For b, c, e and f, each value represents mean ± s.e.m. of 3 samples. **g-i**, Mice were the same as in Fig. 6d. Body weights were monitored (g). After the experiment, each organ was collected and the percentage of each tissue to body weight was plotted (h). H&E analysis was performed in liver and gWAT (i). Each value represents mean ± s.e.m. of 6 mice. Statistical analysis was performed using two-sided unpaired Student's t-tests.

Supplementary Figure 7

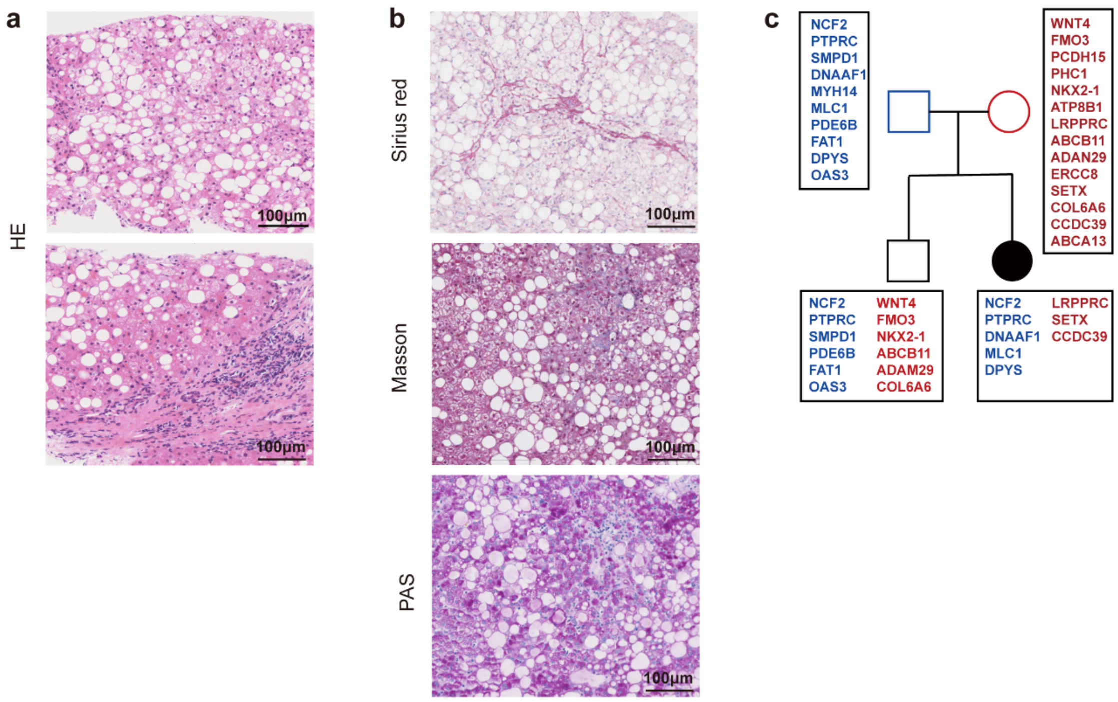

Supplementary Figure 7. Diagnosis of the patient with acquired lipodystrophy.

a-b, Liver biopsy was performed and subjected to H&E analysis (a), or Sirius red, Masson or PAS staining (b). Scale bar, 100 μm. **c**, Whole-exon sequencing of the patient and her family members. The mutations inherited from her father and her mother were colored in blue and red, respectively.

Supplementary Table 1

Primers	Source of Primer sequences
**A. Primers to generate different constructs**	
mFASN-sgRNA	TGGGGTAATGGCCCGGGAGT
mMED20-shRNA1	GCTGATGTACGTGATGCATAA
mMED20-shRNA2	GTACATGGAACTCTTCAACAA
mSNAIL-shRNA1	ATGTGTCTCCCAGAACTATTT
mSNAIL-shRNA2	CCACTCGGATGTGAAGAGATA
mSLUG-shRNA1	GCAGACCCACTCTGATGTAAA
mSLUG-shRNA2	CTCTATGAAAGTTACCCTATA
**Quantitative real-time PCR Primers**	
mAcly	GCCAGCGGGAGCACATC<br>CTTTGCAGGTGCCACTTCATC
mFasn	GCTGCGGAAACTTCAGGAAAT<br>AGAGACGTGTCACTCCTGGACTT
mAcc1	TGGACAGACTGATCGCAGAGAAAG<br>TGGAGAGCCCCACACACA
mAdgre1	CACTTCCAAGATGGGTTAACATCC<br>CTGCCATCAACTCATGATACCCT
mCd11c	CTGGATAGCCTTTCTTCTGCTG<br>GCACACTGTGTCCGAACTC
mIl1b	TAGTCCTTCCTACCCCAATTTCC<br>TTGGTCCTTAGCCACTCCTTC
mIl6	TAGTCCTTCCTACCCCAATTTCC<br>TTGGTCCTTAGCCACTCCTTC
mMed20	AGGTGGAGTATGGCCCTTGT<br>GCATCGTGTCTGTTCCCAAAT
mSnai1	CACACGCTGCCTTGTGTCT<br>GGTCAGCAAAAGCACGGTT
mSnai2	TGGTCAAGAAACATTTCAACGCC<br>GGTGAGGATCTCTGGTTTTGGTA
**Genotyping Primers**	
MED20-flox-F	GGGCAGCCAAGGTCAGTA
MED20-flox-R	AAGTGCTTCCCGTTATTGTGT
AdipoQ-WT-F	CTAGGCCACAGAATTGAAAGATCT
AdipoQ-WT-R	GTAGGTGGAAATTCTAGCATCATCC
AdipoQ-TG-F	ACGGACAGAAGCATTTTCCA
AdipoQ-TG-R	GGATGTGCCATGTGAGTCTG
ERT2-TG-F	TGAAACAGGGGCAATGGTGCG
ERT2-TG-R	CGGAATAGAGTATGGGGGGCTCAG

Supplementary Table 2. Medical index before and after GSH treatment

	Before GSH	After GSH	Reference range
Body weight (kg)	52.4	53.3	N/A
BMI (kg/m^2)	21.0	21.3	18.5-24.0
ALT (U/L)	70.4	34.3	9.0-50.0
AST (U/L)	43.0	29.5	15.0-40.0
TG (mmol/L)	1.76	2.14	0.00-2.25
TC (mmol/L)	3.81	3.68	3.00-5.70
HDL-C (mmol/L)	0.67	0.63	1.03-1.55
LDL-C (mmol/L)	2.72	2.59	2.60-4.10
**Fibroscan**			
CAP (dB/m)	263 (S2)	240 (S1)	N/A
LSM (kPa)	6.3 (F0F1)	6.0 (F0F1)	N/A
**DXA**			
Total body fat (kg)	6.53	6.75	N/A
FMI (kg/ m^2)	2.62	2.71	5.00-9.00
ALM (kg)	17.59	17.26	N/A
ALM/height^2	7.05	6.91	≥5.40
**Fat monitor**			
VAT (cm^2)	5	5	10-100
SAT (cm^2)	78	88	N/A

On top of LANTUS insulin glargine injection (20 u, qn), the patient was treated with GSH (0.4 g, tid) from Nov. 2021 to May 2022. Different parameters were monitored before and after GSH treatment. BMI, body mass index; ALT, alanine transaminase; AST, aspartate aminotransferase; TG, triglyceride; TC, total cholesterol; HDL-C, high-density lipoprotein cholesterol; LDL-C, low-density lipoprotein cholesterol; CAP, controlled attenuation parameter; LSM, liver stiffness measurement; DXA, dual-energy x-ray absorptiometry; FMI, fat mass index; ALM, appendicular lean mass, also known as the lean mass of the upper and lower limbs; VAT, visceral fat; SAT, subcutaneous fat. S1 and S2 in CAP indicate steatosis grade 1 and steatosis grade 2, respectively.

Supplementary Table 3

Abbreviation	Meaning
FASN	Fatty Acid Synthase
ROS	Reactive Oxygen Species
LDH	Lactate Dehydrogenase
NEC-1	Necrostatin-1
Lip-1	Liproxstatin-1
BHA	Butyhydroxyanisole
GSH	L-Glutathione Reduced
SVFs	Stromal Vascular Fractions
Acly	ATP-Citrate Lyase
Acc1	Acetyl-CoA Carboxylase
iWAT	Inguinal White Adipose Tissue
gWAT	Gonadal White Adipose Tissue
BAT	Brown Adipose Tissue
NRTI	Nucleoside Analog Reverse Transcriptase Inhibitors
BMI	Body Mass Index
FMI	Fat Mass Index
NASH	Nonalcoholic Steatohepatitis
CAP	Controlled Attenuation parameter
MRI-PDF	Magnetic Resonance Imaging-derived Proton Density Fat Fraction
