# Supplementary material for: Surplus fatty acid synthesis increases oxidative stress in adipocytes and induces lipodystrophy
Source: Nat Commun. 2024 Jan 2;15:133. doi: 10.1038/s41467-023-44393-7 (PMC10761979; doi:10.1038/s41467-023-44393-7)
Supplement: Supplementary file 3 — Reporting Summary [file 41467_2023_44393_MOESM3_ESM.pdf]

nature portfolio

Corresponding author(s): Tong-Jin Zhao

Last updated by author(s): Dec 11, 2023

Reporting Summary

Nature Portfolio wishes to improve the reproducibility of the work that we publish. This form provides structure for consistency and transparency in reporting. For further information on Nature Portfolio policies, see our Editorial Policies and the Editorial Policy Checklist.

Statistics

For all statistical analyses, confirm that the following items are present in the figure legend, table legend, main text, or Methods section.

n/a	Confirmed	
☐	☒	The exact sample size (*n*) for each experimental group/condition, given as a discrete number and unit of measurement
☐	☒	A statement on whether measurements were taken from distinct samples or whether the same sample was measured repeatedly
☐	☒	The statistical test(s) used AND whether they are one- or two-sided<br>*Only common tests should be described solely by name; describe more complex techniques in the Methods section.*
☒	☐	A description of all covariates tested
☒	☐	A description of any assumptions or corrections, such as tests of normality and adjustment for multiple comparisons
☐	☒	A full description of the statistical parameters including central tendency (e.g. means) or other basic estimates (e.g. regression coefficient) AND variation (e.g. standard deviation) or associated estimates of uncertainty (e.g. confidence intervals)
☐	☒	For null hypothesis testing, the test statistic (e.g. *F*, *t*, *r*) with confidence intervals, effect sizes, degrees of freedom and *P* value noted<br>*Give P values as exact values whenever suitable.*
☒	☐	For Bayesian analysis, information on the choice of priors and Markov chain Monte Carlo settings
☒	☐	For hierarchical and complex designs, identification of the appropriate level for tests and full reporting of outcomes
☒	☐	Estimates of effect sizes (e.g. Cohen's *d*, Pearson's *r*), indicating how they were calculated

Our web collection on statistics for biologists contains articles on many of the points above.

Software and code

Policy information about availability of computer code

Data collection	Zeiss LSM-880+ confocal microscopy was used to take fluorescent images. MiniChemi 910 Chemiluminescence Imaging System (Beijing Sage Creation) was used for collection of Western blot images. the fluorescent intensity of DCFDA was quantified on a Tecan Spark plate reader. Evident Olympus VS200 microscope was used to visualize the slides of immunohistochemistry of p-MLKL.
Data analysis	ZEN2.3 and Image J were used to quantify fluorescent intensity and colocalization. EXCEL2O1O was used to analyze data and student's t test. The raw files of Mass Spectrometry were analyzed by OS-MQ software (AB SCIEX).

For manuscripts utilizing custom algorithms or software that are central to the research but not yet described in published literature, software must be made available to editors and reviewers. We strongly encourage code deposition in a community repository (e.g. GitHub). See the Nature Portfolio guidelines for submitting code & software for further information.

Data

Policy information about availability of data

All manuscripts must include a data availability statement. This statement should provide the following information, where applicable:

- Accession codes, unique identifiers, or web links for publicly available datasets
- A description of any restrictions on data availability
- For clinical datasets or third party data, please ensure that the statement adheres to our policy

All data generated or analyzed during this study are included in this published article and its supplementary information files.

Research involving human participants, their data, or biological material

Policy information about studies with human participants or human data. See also policy information about sex, gender (identity/presentation), and sexual orientation and race, ethnicity and racism.

Reporting on sex and gender	female
Reporting on race, ethnicity, or other socially relevant groupings	Asian
Population characteristics	Generalized lipodystrophy
Recruitment	The patient came to the hospital as she did not feel well. She was diagnosed with generalized lipodystrophy. There is only one patient used in the study, as lipodystrophy is a rare disease.
Ethics oversight	The study protocol was reviewed and approved by the Human Ethics Committee of the First Affiliated Hospital of Nanjing Medical University (2019-SR-122)

Note that full information on the approval of the study protocol must also be provided in the manuscript.

Field-specific reporting

Please select the one below that is the best fit for your research. If you are not sure, read the appropriate sections before making your selection.

☒ Life sciences ☐ Behavioural & social sciences ☐ Ecological, evolutionary & environmental sciences

For a reference copy of the document with all sections, see nature.com/documents/nr-reporting-summary-flat.pdf

Life sciences study design

All studies must disclose on these points even when the disclosure is negative.

Sample size	No sample size calculation was performed. According to our experience, 3 samples were used for cell culture experiments, and at least 6 mice in each group were used for animal studies.
Data exclusions	No data was excluded.
Replication	All the experiments were repeated at least twice, and similar results were obtained. The number of replicates was stated at the end of each figure legend.
Randomization	The samples were numbers in Arabic numbers instead of real names to avoid any bias during the experiments.
Blinding	Investigators were blind to group allocation.

Reporting for specific materials, systems and methods

We require information from authors about some types of materials, experimental systems and methods used in many studies. Here, indicate whether each material, system or method listed is relevant to your study. If you are not sure if a list item applies to your research, read the appropriate section before selecting a response.

Materials & experimental systems

n/a	Involved in the study	
☐	☒	Antibodies
☐	☒	Eukaryotic cell lines
☒	☐	Palaeontology and archaeology
☐	☒	Animals and other organisms
☒	☐	Clinical data
☒	☐	Dual use research of concern
☒	☐	Plants

Methods

n/a	Involved in the study	
☒	☐	ChIP-seq
☒	☐	Flow cytometry
☒	☐	MRI-based neuroimaging

Antibodies

Antibodies used	anti-β-Actin (1:1,000, Protein Tech, 60008-1-Ig), anti-MED20 (1;1000, Protein Tech, 17598-1-AP), anti-GAPDH (1:5000, CST, 5174s), anti-MLKL (1:1,000, Abcam, ab184718), anti-P-MLKL (1:1,000; Abcam, ab187091), anti-Cleaved Caspase-3 (1:1,000, CST, 4190), anti-

	Acetyl-CoA Carboxylase1 (1:1000, CST, 4190), anti-ACLY (1:1000, CST, 13390), anti-RIPK3 (1:1000, ABclonal, A5431), anti-RIPK1 (1:1000, ABclonal, A7414), anti-P-RIPK3 (1:1000, Abcam, ab205421), anti-P-RIPK1 (1:1000, ABclonal, AP1230), anti-C/EBPα (1:1000, CST, 8178S), anti-PPARγ (1:1000, Santa Cruz, sc-7273), anti-CD36 (1:1000, Sino Biological Inc, 80263-T48), anti-Perilipin 1 (1:1000, CST, 9349s), anti-Galectin3 (1:1000, Protein Tech, #60207-1-Ig), anti-FASN(1:1000, CST, 3180) and Goat anti-Rabbit IgG (H+L) (1:10000, Jackson Immuno Research, 118578)
Validation	anti-β-Actin (Protein Tech, 60008-1-Ig), validated by WB in various tissues and cell lines; anti-MED20 (Protein Tech, 17598-1-AP), validated by WB in HeLa cells. anti-GAPDH (CST, 5174s), validated by WB in HeLa cells, NIH/3T3, COS-7 and C6 cells;anti-MLKL (ab184718), validated by WB in HAP1 cells, HeLa cells, Huvec cells, IF in HT-29 cells, IHC in paraffin-embedded Human colonic adenocarcinoma tissue; anti- P-MLKL (phospho 5358, Abcam, ab187091), validated by WB in HT-29 cells, human hepatocyte cell, IHC in Human melanoma tissue, Human skin tissue; anti-Cleaved Caspase-3 (CST, 9661), validated by WB in HeLa cells, NIH/3T3 and C6 cells, IHC in human tonsil, Jurkat cells, mouse embryo, IF in HT-29 cells, flow cytometry in Jurkat cells; anti-Acetyl-CoA Carboxylase I (CST, 4190), validated by WB in HeLa cells, Hep G2cells, 293T cells, NBT-II cells; anti-ACLY (CST, 13390), validated by WB in MCF7cells, HeLa cells, Hep G2 cells, mIMCD-3 cells; anti-RIPK3 (ABclonal, A5431), validated by WB in L929 cells, IF in C6 cells, NIH/3T3 cells, PC-12 cells and HT-29 cells, IHC in human liver cancer and mouse pancreas; anti-RIPK1 (ABclonal, A7414), validated by WB in various cell lines, IF in NIH/3T3 cells, IHC in mouse colon; anti-P-RIPK3 (Abcam, ab205421), validated by WB in mouse pancreas, kidney and small intestine tissues; anti-P-RIPK1 (ABclonal, AP1230), validated by WB in HT-29 cells; anti-C/EBPα (CST, 8178S), validated by WB in Hep G2 and LNCaP cells, IF in THP-1 and Jurkat cells, IHC in mouse lung; anti-PPARγ (Santa Cruz, sc-7273), validated by WB in HeLa, Jurkat, U-937, THP-1 and MDA-MB-231 cells, IF in Jurkat cells; nti-CD36 (Sino Biological Inc, 80263-T48), validated by WB in Mouse heart tissue; anti-Perilipin I (CST, #9349s), validated by WB in human pre-adipocytes and adipocytes, IP in 3T3-L1 cells, IHC in Human breast carcinoma, mouse brown fat, mouse kidney, mouse prostate, mouse testis, IF in mouse brown adipose tissue, 3T3-L1 cells; anti-Galectin3 (Protein Tech, #60207-1-Ig), validated by WB in various tissues and cell lines, IHC in human various tissues, IF in HeLa cells, human thyroid cancer tissue, MCF-7 cells, flow cytometry in HeLa cells; anti-FASN(CST, 3180), validated by WB in various cell types, IHC in breast carcinoma, IF in HeLa cells.

Eukaryotic cell lines

Policy information about cell lines and Sex and Gender in Research

Cell line source(s)	All the cell lines were originally obtained from ATCC. HEK293T cells were obtained from Brown&Goldstein's laboratory at UT Southwestern Medical Center.
Authentication	None of the cell lines were authenticated.
Mycoplasma contamination	The cell lines were tested negative for mycoplasma.
Commonly misidentified lines (See ICLAC register)	No.

Animals and other research organisms

Policy information about studies involving animals; ARRIVE guidelines recommended for reporting animal research, and Sex and Gender in Research

Laboratory animals	Med20f/f mice were previously generated by our laboratory, and they were bred with an AdipoQ-Cre mice to generate adipocyte-specific Med20 knockout mice (Med20-AKO, male, 2-14 weeks old); Med20f/f mice were also crossed with Gt(ROSA) 26Sortm1(cre/ERT2)Tyj mice to generate inducible Med20 knockout mice(male, 4 weeks old), which was used for isolation of primary stromal vascular fractions; and C57BL6J mice (male, 5-6 weeks old) were purchased from GemPharmatech (Nanjing, China).
Wild animals	no wild animals were used in the study.
Reporting on sex	All the mice strains used in the study were male.
Field-collected samples	The study did not involve samples collected from the field.
Ethics oversight	All animal studies were performed with the approval of the Institutional Animal Care and Research Advisory Committee at Fudan University and Xiamen University.

Note that full information on the approval of the study protocol must also be provided in the manuscript.

Plants

Seed stocks	*Report on the source of all seed stocks or other plant material used. If applicable, state the seed stock centre and catalogue number. If plant specimens were collected from the field, describe the collection location, date and sampling procedures.*
Novel plant genotypes	*Describe the methods by which all novel plant genotypes were produced. This includes those generated by transgenic approaches, gene editing, chemical/radiation-based mutagenesis and hybridization. For transgenic lines, describe the transformation method, the number of independent lines analyzed and the generation upon which experiments were performed. For gene-edited lines, describe the editor used, the endogenous sequence targeted for editing, the targeting guide RNA sequence (if applicable) and how the editor was applied.*
Authentication	*Describe any authentication procedures for each seed stock used or novel genotype generated. Describe any experiments used to assess the effect of a mutation and, where applicable, how potential secondary effects (e.g. second site T-DNA insertions, mosiacism, off-target gene editing) were examined.*
